# Protective Effects of Shrimp Peptide on Dextran Sulfate Sodium-Induced Colitis in Mice

**DOI:** 10.3389/fnut.2021.773064

**Published:** 2021-11-24

**Authors:** Xingwei Xiang, Qihong Jiang, Wan Shao, Jinhong Li, Yufang Zhou, Lin Chen, Shanggui Deng, Bin Zheng, Yufeng Chen

**Affiliations:** ^1^College of Food Science and Technology, Zhejiang University of Technology, Hangzhou, China; ^2^Key Laboratory of Marine Biological Resources Innovation and Development of Zhejiang Province, Hangzhou, China; ^3^National R&D Branch Center for Pelagic Aquatic Products Processing (Hangzhou), Hangzhou, China; ^4^Food and Pharmacy College, Zhejiang Ocean University, Zhoushan, China; ^5^Zhejiang Marine Development Research Institute, Zhoushan, China; ^6^Institute of Sericultural and Tea, Zhejiang Academy of Agricultural Sciences, Hangzhou, China

**Keywords:** shrimp peptides, dextran sulfate sodium (DSS), ulcerative colitis, inflammatory bowel disease, intestinal flora

## Abstract

Inflammatory bowel disease, an intestinal relapsing inflammatory disease, not only impairs gastrointestinal function but also increases the chances of developing colon cancer. Currently, the effects of shrimp peptide (SP) in mice model of ulcerative colitis (UC) are still unclear. In particular, it is uncertain whether SP affects the gut flora with UC mice. In this study, we investigated the anti-inflammatory effects of SP on a dextran sulfate sodium (DSS)-induced mouse model of UC. Firstly, the molecular weight of SP was mainly distributed in the range of 180–1,000 Da (61.95% proportion), and the amino acid composition showed that SP contained 17 amino acids, of which, the essential amino acids accounted for 54.50%. *In vivo*, oral SP significantly attenuated the severity of colitis, such as diarrhea, weight loss, and rectal bleeding. Furthermore, treatment with SP remarkably ameliorated intestinal barrier integrity, thus lowering the levels of the inflammatory cytokines and ameliorating antioxidant indices and intestinal injury indicators in the serum and colon. Lastly, the cecal contents were used to sequence and analyze the 16S rRNA genes of bacteria. Results suggested that treatment with SP could restore the balance of intestinal flora in modeled mice by regulating the abundance of pathogenic and beneficial bacteria. Furthermore, SP could significantly improve intestinal flora dysfunction in mice with UC. In summary, our findings show that SP has a prophylactic and therapeutic effect in UC *in vivo*, thereby highlighting its broad medicinal applications.

## Introduction

Inflammatory bowel disease (IBD) is a chronic recurrent inflammatory disease of the gastrointestinal tract that includes Crohn's disease and ulcerative colitis (UC). Among these conditions, UC is a recurrent inflammatory chronic disease that most frequently affects the colon in adults, leading to weight loss, abdominal pain, diarrhea, and rectal hemorrhage (1). The etiology of chronic IBD is not completely understood but may be influenced by genetic, immunological, and environmental factors. Particularly, the protective function of the intestinal barrier is closely related to immune cells and cytokines. Previous studies have reported that activated immune cells can aggravate IBD by damaging the intestinal barrier function by cytokine secretion (2). Meanwhile, clinical studies have shown that oxidative stress signals participate in and promote IBD responses at multiple functional levels. Oxidative stress damages the mucosal layer of the gastrointestinal (GI) tract and leads to bacterial invasion, which in turn stimulates the immune response and initiates IBD (3).

The pro-oxidant-antioxidant level involves a complex balance, and dysregulation or disturbance of this balance leads to an oxidative stress response. This phenomenon occurs in all organisms. Increased oxidative stress can cause cellular and tissue damage, which results in the inability to resolve the inflammatory response, thereby leading to a chronic inflammatory state (4). Several pro-inflammatory cytokines, such as tumor necrosis factor (TNF)-α and interleukin (IL)-6, affect the systemic inflammatory reaction either directly or indirectly (5). Gut microbiota is associated with the occurrence and development of UC and have been recognized as an “environmental factor” and are accompanied by an impairment in intestinal mucosal barrier function, imbalance in intestinal microbiota, and abnormality of metabolites (6, 7). Specifically, adverse changes in the composition of the intestinal flora, changes in the main *Firmicutes*/*Bacteroidetes* ratio, and decreases in the diversity and richness of intestinal microbial communities can lead to systemic intestinal inflammation (8). Numerous studies have suggested that the inclusion of probiotics or prebiotics in the diet can effectively improve the structural composition of intestinal flora in patients with UC. Thus, protecting and enhancing the gut microbiota through a suitable diet is an effective and low-risk disease-treatment strategy.

In recent years, natural active substances extracted from animals and plants have been widely researched by scholars worldwide owing to their safety and efficacy. Some biologically active peptides can repair the intestinal barrier and treat intestinal inflammation (9–11). The marine shrimp-processing industry has developed rapidly in China. Chinese pipe whip shrimps (*Solenocera crassicornis*), commonly known as red shrimp, are rich in proteins and minerals and have economic importance as marine shrimp (12). Large quantities of shrimp components including its head and shell are often discarded. The by-products from shrimp processing are abundant in amino acids, and the ratio of amino acids to proteins is very close to that of human muscle (13), rendering them a good protein resource. The use of by-products from shrimp processing in the preparation of active peptides can be of significance for improving resource utilization of bulk seafood.

Shrimp peptide (SP) has biological activities such as hepatoprotective (14), antioxidant (15, 16), anti-inflammatory (17), and antibacterial effects (18, 19). However, it is not known whether Chinese pipe whip SP can protect dextran sulfate sodium (DSS)-induced mice with UC. Moreover, it is unclear whether SP can regulate the gut flora of mice with UC. In this study, we comprehensively illustrated the anti-inflammatory effect and plausible mechanism of action of SP in a DSS-induced mouse model of UC. The effect of SP on intestinal barrier integrity, inflammatory cytokines, antioxidant indices, and intestinal flora in a DSS-induced mouse model of UC was determined. Our findings suggest the development of SP as a functional food as a new strategy to prevent inflammation-related intestinal illnesses.

## Materials and Methods

### Chemicals and Reagents

SP was produced in our laboratory using *Solenocera crassicornis* by-products obtained from Zhoushan Yueyang Food Co., Ltd (Zhoushan, China). DSS [molecular weight (MW) 36–50 KDa] was purchased from MP Biomedicals (Santa Ana, CA, USA). Standard food for mice was obtained from Suzhou Shuangshi Experimental Animal Feed Technology Co., Ltd (Suzhou, China). ELISA kits for IL-6 and TNF-α were purchased from the Wuhan Boshide Biological Engineering Co. LTD (Wuhan, China); ELISA kits for DAO and LPS were purchased from Wuhan Huamei Biological Company (Wuhan, China); ELISA kits for SOD and GSH-Px were purchased from Nanjing Jiancheng Institute of Biological Engineering (Nanjing, China). Primers were designed using Primer Premier 5.0 and synthesized by Sangon Bioengineering (Shanghai, China) Co. Ltd. All other chemicals, solvents, and reagents were of analytically pure grade.

### Preparation of SP

The heads of red shrimp were mixed with 95% ethanol (1:10, w/v) and stirred and degreased for 8 h at 25°C. The filtered residue was dried at 45°C. Next, distilled water (1:2, w/v) was added to the residue and the pH was adjusted to 7.2. Enzymatic hydrolysis at 55°C was performed using 2% alkaline protease for 5 h. The enzyme was inactivated at 90°C for 20 min, and the supernatant was filtered and collected. The hydrolysates were separated using an inorganic 0.5-μm pore size ceramic membrane. The collected permeates were successively passed through 1,000 and 250 Da ultrafiltration membranes, and the permeate and the intercepted solution were collected to obtain 250–1,000 Da components, which were freeze-dried to obtain SP.

### Determination of MW Distribution

Cytochrome c (MW: 12,355 Da), aprotinin (MW: 6,511 Da), bacitracin (MW: 1,422 Da), and ethylene-ethylene-tyrosine-arginine (MW: 451 Da) were weighed and dissolved in the mobile phase to yield standard molecular weight solutions of 0.2 mg/mL. The sample (100 mg) was weighed into a volumetric flask, diluted to 10 mL with the mobile phase, shaken, and filtered through a microporous membrane (0.45 μm) to remove impurities and facilitate sample injection. The MW distribution of SP was determined using high-performance gel filtration chromatography under the following conditions: gel column: TSK gel G2000 SWXL analytical column (7.8 × 300 mm, 5 μm); mobile phase: acetonitrile/water/trifluoroacetic acid (40:60:0.05, v/v/v); flow rate: 0.5 mL/min; detection wavelength: 220 nm; column temperature: 30°C; injection volume: 20 μL.

### Determination of Amino Acid Composition

About 1 g of the sample was weighed and dissolved in 5% trichloroacetic acid. The sample was kept at a constant volume of 25 mL and mixed using ultrasound for 20 min at room temperature. After standing for at least 2 h, the supernatant was filtered using a double-layer filter paper. About 400 μL of the supernatant was refiltered using a 0.22-μm microporous membrane and the amino acid composition was determined using OPA pre-column derivatization and reversed-phase HPLC (Agilent, UK) with UV detection. The operational conditions were as following: gradient dilution with 80.9 mmol/L sodium acetate-methanol-acetonitrile (1:2:2, v/v; pH 7.2). The flow rate was 1.0 mL/min; column temperature was 40°C; proline was detected at 262 nm and other amino acids was detected at 338 nm detection wavelength with UV detector, and. The amino acid content was quantified by external standard method.

### Experimental Animals and Establishment of the DSS-Induced Colitis Model

Based on our previously reported method (20), in this study, 32 four-week-old male ICR mice were purchased from Shanghai Slack Laboratory Animal Co., Ltd. All mice were fed a standard diet for 1 week in an animal room and were subjected to a 12 h/12 h dark/light cycle, a temperature of 24 ± 1°C, and humidity of 60 ± 5%. At the end of the adaptation period, the mice were randomly divided into 4 groups (8 mice per group). In order to eliminate the influence of differences between cages on intestinal flora, the same cage is a simple and common method to homogenize the microbial community, but the high number of mice in each cage will lead to stress response and affect the test results. Therefore, in order to ensure the representativeness of the samples, mice in each group were randomly divided into 2 cages (4 mice in each cage) and placed in SPF room. Mice in the control group were provided normal water during the experimental period. From the 15th day until the end of the experiment, mice in both the DSS and SP groups were provided drinking water containing 3.5% DSS (treatment for 8 days). Mice in the SP groups were administered SP extract (300 and 600 mg/kg/day) from day 1 to the end of the experimental period on day 22. All drugs were administered via the intragastric route once daily during the experiment. The body weights and disease activity index (DAI) of mice were recorded daily and routinely (Table 1). The procedure followed in this study was consistent with the ethical standards set by the Animal Experiment Committee of the unit in charge (approval number: 2019003).

**Table 1:** Disease activity index (DAI) score.

**Score**	**Weight loss**	**Stool consistency change**	**Bleeding**
0	None	None	None
1	0–5%	Loose stool	Trace of fecal occult blood
2	5–10%		Mild occult blood
3	10–20%	Diarrhea	Obvious occult blood
4	>20%		Severe bleeding

### Sample Collection

After the experiment, Mice were euthanized by cervical dislocation. Blood was collected from the eyeball and centrifuged for 10 min at 4°C and 3,500 rpm. The serum was separated and stored in a refrigerator at −80°C until later use. Mice were dissected and the intestine was isolated to remove the mesentery. The colon length was recorded and then cut off 2 cm above the anus. The most obvious lesion tissue was placed in 4% paraformaldehyde to prepare sections for histopathological analysis. The rest of the intestinal tubes were stored at −80°C.

### Hematoxylin and Eosin Staining and Histological Scoring

Hematoxylin and Eosin (H&E) staining was performed according to a previously described method (1). Briefly, the middle portions of colon tissues were excised and immobilized in 10% (100 g/L) formalin for 24 h, followed by dehydration with gradient ethanol. Next, the colonic tissues were made transparent with xylene, embedded in paraffin, sliced into 5-μm-thick sections, stained with H&E, and observed using a DP-72 microscope (Olympus, Tokyo, Japan) to assess the morphometry of intestinal epithelial cells, neutrophil density, loss of crypt glands, and lymphocyte infiltration.

### Intestinal Morphology in Mice With Colitis

To observe the ultrastructure of tissues, the collected colon tissues were sliced into several pieces, fixed with 2.5% glutaraldehyde at 4°C, and then fixed again with 1% osmium tetroxide. Next, the samples were dehydrated using ethanol solutions of different concentrations. Subsequently, a portion of the treated colon samples was affixed to the stage and gold plated for scanning electron microscopy (SEM). Another portion of the sample was impregnated using epoxy resin, sliced into ultrathin sections, and stained with uranium acetate and aluminum citrate for transmission electron microscopy (TEM) (21). The tight junctions (TJ), microvilli, and other structures of colon epithelial cells were observed using a computer-aided image-analysis system following SEM and TEM.

### Determination of Serum and Intestinal Tissue-Related Indices in Colitis Mice

The separated serum was divided into Eppendorf tubes for cryopreservation, placed at room temperature, and processed according to the instructions on the kit. Superoxide dismutase (SOD), glutathione reductase (GSH-Px), IL-6, TNF-α, diamine oxidase (DAO), and lipopolysaccharide (LPS) levels in mouse serum were determined.

Colon tissues (100 mg) from different treatment groups were treated with 1 mL of sterile physiological saline. The intestines were ground to mucus using an electric homogenizer and centrifuged for 10 min at 4,000 r/min in a precooled apparatus at 4°C. The supernatant was divided and stored in a refrigerator at −80°C. A bicinchoninic acid kit was used to determine the protein concentration and GSH-Px, SOD, TNF-α, IL-6, DAO, and LPS levels in the intestinal tract were determined.

### Total DNA Extraction and Illumina Miseq Sequencing of Fecal Samples

Total bacteriological DNA was obtained from fecal samples of each group of mice following the instructions in the MOBIO PowerSoil DNA Isolation kit (MOBIO, United States) (22). DNA integrity was determined using agarose gel electrophoresis, and a QubitTM3 Fluorometer (Invitrogen, USA) was used to determine the DNA concentration in each sample. Next, the primers 341F (5′-ACTCCTACGGGAGGCAGCAG-3′) and 806R (5′-GGACTACHVGGGTWTCTAAT-3′) were used to amplify the V3–V4 high variant region of the 16S rRNA gene using PCR. The amplification products of PCR were purified using Ampure XP magnetic beads (AGENCOURT, Beckman Coulter, US) to remove the non-specific amplification products. An Agilent 2100 Bioanalyzer (Agilent DNA 1000 Reagents) was used to determine the average molecular length of the amplified library and quantify DNA libraries using real-time quantitative PCR. Lastly, the Illumina miseq platform was used to complete the sequencing of the qualified libraries. To ensure accurate and reliable results for subsequent analysis, DNA sample testing, library construction and quality control were performed on the raw sequencing data with subsequent sequencing consigned to Shanghai Meiji Biomedical Technology Co., Ltd. The number of reads per sample is 40–50,000, and rarefaction was used to reflect the microbial diversity of each sample at different sequencing volumes, indicating whether the amount of sequencing data for the sample is reasonable. In addition, the number of species common and unique species (OTU) in multiple groups or samples is calculated by Venn diagrams, which allow OTU classification of all sequences according to different levels of similarity, and usually perform bioinformatic statistical analysis of OTUs at 97% similarity level. PCoA is used to reflect the differences of multiple data sets on a two-dimensional coordinate plot using variance decomposition, with the axes taking the two eigenvalues that best reflect the differences between samples. The original sequencing data were analyzed on the Meiji Biocloud website.

### Statistical Analysis

GraphPad Prism 10.01 was used for statistical analysis of the data, which are expressed as mean ± standard deviation. One-way ANOVA analysis with *post-hoc* independent samples *t*-tests was used for multiple comparisons to test the significance of differences. Differences were considered statistically significant at *P* < 0.05.

## Results

### Characterization of SP

**Figure 1:**
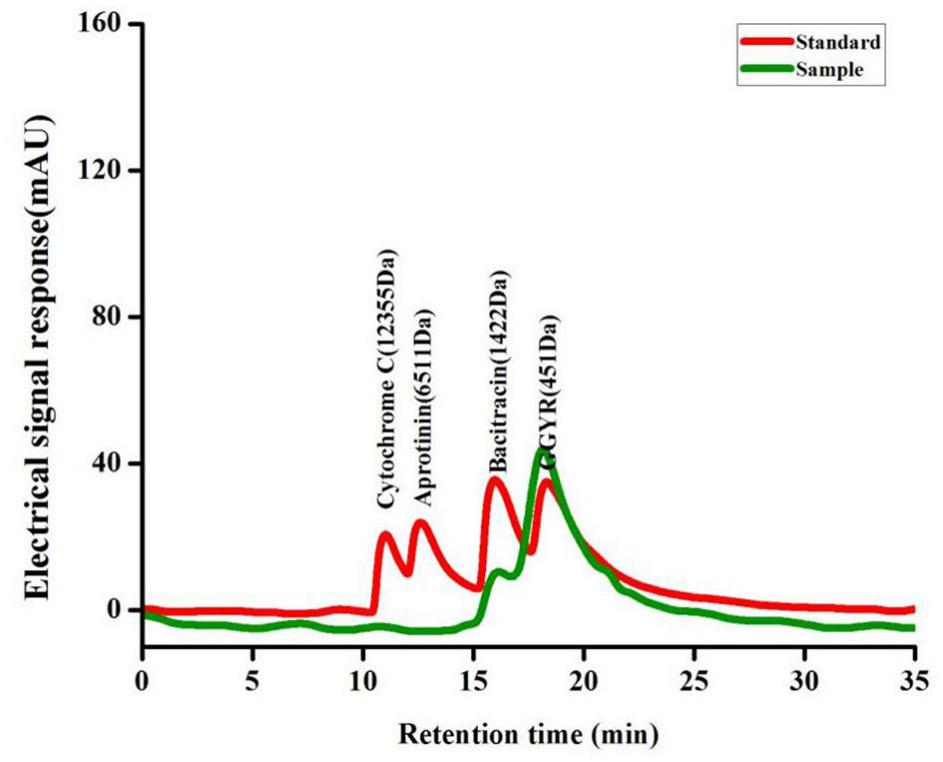
The HPLC chromatograms of the standard molecular weight samples and the SP.

MW distribution is an important parameter for the evaluation of proteolytic products as it may affect the antioxidant activity and other functional properties. The MW distribution of SP was determined using HPLC under the same chromatographic conditions as those used for standard samples (Figure 1). The MW distribution of SP was mainly below 1,000 Da, and the distribution range of 180–500 and 500–1,000 Da accounted for 35.20 and 26.75%, respectively. Studies show that short peptides with low MWs tend to be better absorbed; thus, SP being a small MW peptide could be easily digested and absorbed.

The amino acid composition of SP is shown in Table 2. SP has 17 amino acids, among which, the essential amino acid content is 54.50%. Lysine, leucine, glutamic acid, and arginine are abundant and account for 11.03, 14.63, 11.65, and 15.13%, respectively. Besides, methionine (Met), threonine (Thr), tryptophan (Trp), and histidine (His) have been identified as antioxidant amino acids. Moreover, their derivatives and peptides containing these amino acids exert antioxidant effects.

**Table 2:** The amino acid composition of SP.

**Amino acid**	**Abbreviation**	**Ratio (g/100 g)**
Aspartic acid	Asp	7.57549e-1
Glutamic acid	Glu	2.20
Serine	Ser	1.05743e-1
Histidine	His	2.91005e-1
Histidine	Gly	4.12054e-1
Threonine	Thr	6.79615e-1
Arginine	Arg	2.86
Alanine	Ala	6.12500e-1
Tyrosine	Tyr	1.20
	Cys-s	1.56135e-2
Valine	Val	1.18
Methionine	Met	6.43734e-1
Phenylalanine	Phe	1.87
Isoleucine	Ile	1.09
Leucine	Leu	2.77
Lysine	Lys	2.09
Proline	Pro	1.43503e-1
	Hyp	4.67176e-2

### SP Ameliorates the Inflammatory Symptoms of DSS-Induced Colitis in Mice

**Figure 2:**
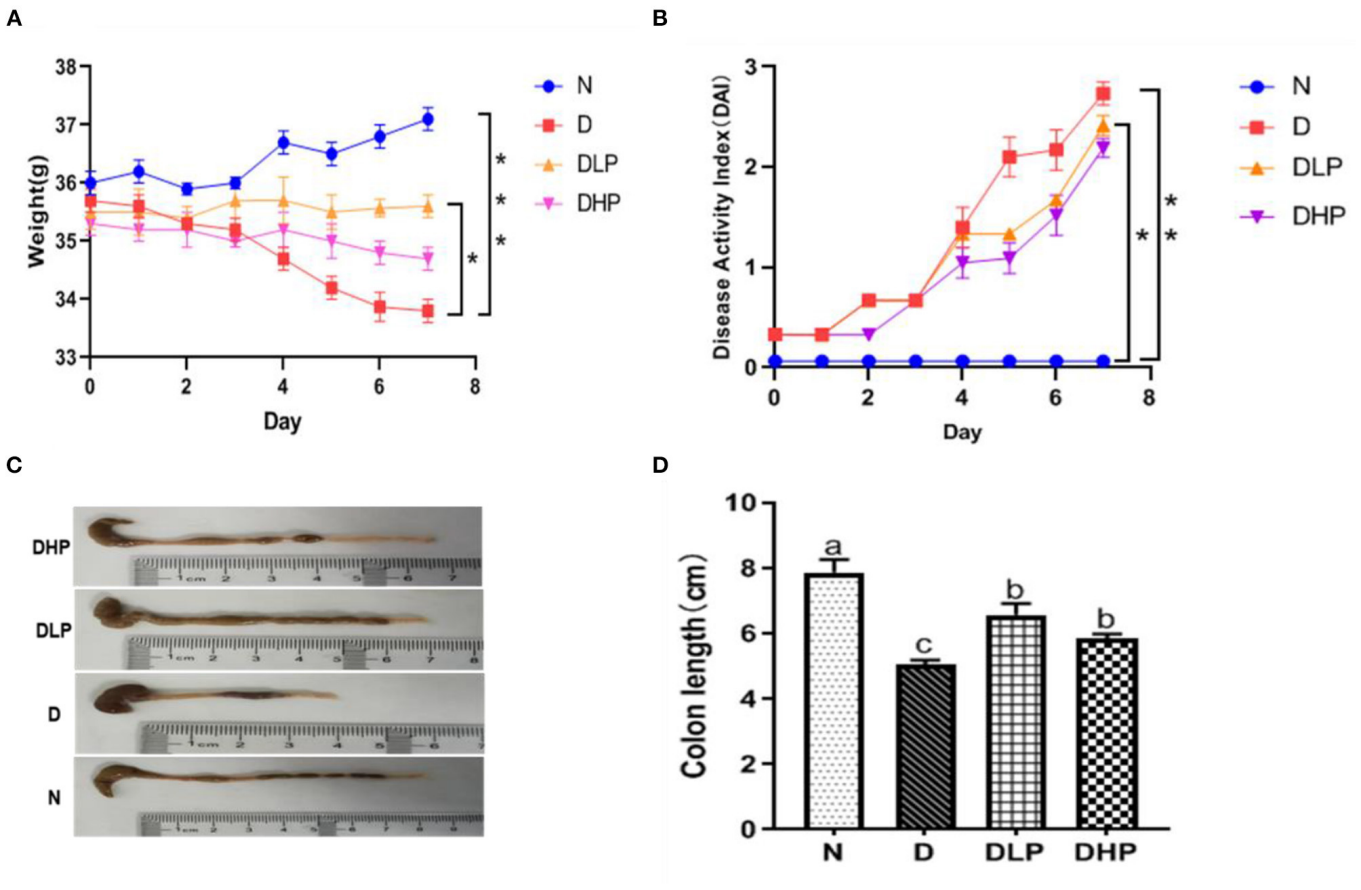
Effect of SP on body weight change (%) **(A)**, Disease activity index **(B)**, Macroscopic appearances of colons **(C)** and Colon length **(D)** of colitis in mice. Different letters were presented with significant (*P* < 0.05). N, the control group; D, the DSS-treated group; DLP, DSS + 300 mg/kg SP; DHP, DSS + 600 mg/kg SP.

Mice in the normal control group had normal stools, shiny fur, sensitive reaction, and normal activity. After DSS induction, the model mice exhibited different degrees of pathological symptoms, including diarrhea, bloody stools, darkening of fur, loss of appetite, and laziness. Mice in the low- and high-dose SP groups also showed changes in stool characteristics after modeling, such as diarrhea and bloody stools, and the symptoms were significantly less intense than those observed in the DSS-treated group, suggesting better activity and diet status, and a reduction in diarrhea and bloody stools. As shown in Figures 2A,B, during the modeling process, mice in the control group showed no overall decrease in body weight; however, mice in the DSS-treated group showed a significant decrease in body weight (*P* < 0.05) and had higher DAI scores compared with those in the blank control group. After treatment with low-dose and high-dose SP, the loss of body weight in mice with DSS-induced colitis was significantly inhibited and DAI scores were also found to decrease. It was worth noting that low-dose SP (300 mg/kg) showed a better effect in maintaining body weight and DAI score compared with that observed using high-dose SP (600 mg/kg). Furthermore, as shown in Figures 2C,D, the colon length was significantly shorter in mice in the DSS group compared with those in the blank control group (*P* < 0.05). Moreover, the colon lengths of mice in the low-dose and high-dose SP groups were significantly longer (*P* < 0.05) compared with those of mice in the DSS group. The colon lengths of mice in the low-dose and high-dose SP groups were not significantly different (*P* > 0.05). Collectively, these results indicated that treatment with SP could improve the weight decrease and shortened colon length and reduce the DAI scores in mice with DSS-induced UC.

### SP Mitigated the Histopathology of DSS-Induced Colitis Mice

**Figure 3:**
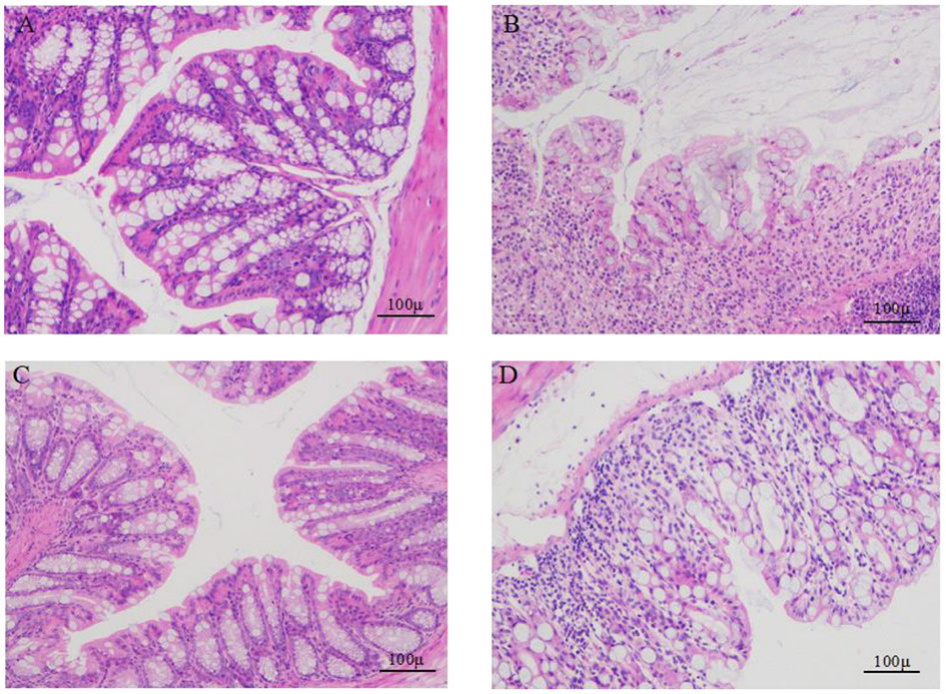
Comparison of the colon morphology of mice. **(A)** colons of control mice; **(B)** colons of DSS treated mice; **(C)** colons of DSS and SP (300 mg/kg/day) treated mice; **(D)** colons of DSS and SP (600 mg/kg/day) treated mice.

As seen in Figure 3, HE staining of the colon tissue showed that the tissues from the normal control group (Figure 3A) had clear colon walls, intact epithelia, neatly arranged glands, normal crypts, abundant goblet cells, and no inflammatory cell infiltration. The colonic epithelia in the DSS model group (Figure 3B) samples appeared necrotic and shedding. The intestinal wall was thin, glands were messy, ulcer symptoms were severe, and a great extent of inflammatory cell infiltration was found in the mucosal muscle layer. The colonic mucosa and intestinal villi in the tissue samples from the low-dose (Figure 3C) and high-dose SP groups (Figure 3D) appeared less damaged than those in the DSS model group, and the colonic mucosa structure was intact in the SP-treated groups. Furthermore, the improvement achieved after low-dose SP treatment was better than that achieved using the high dose.

**Figure 4:**
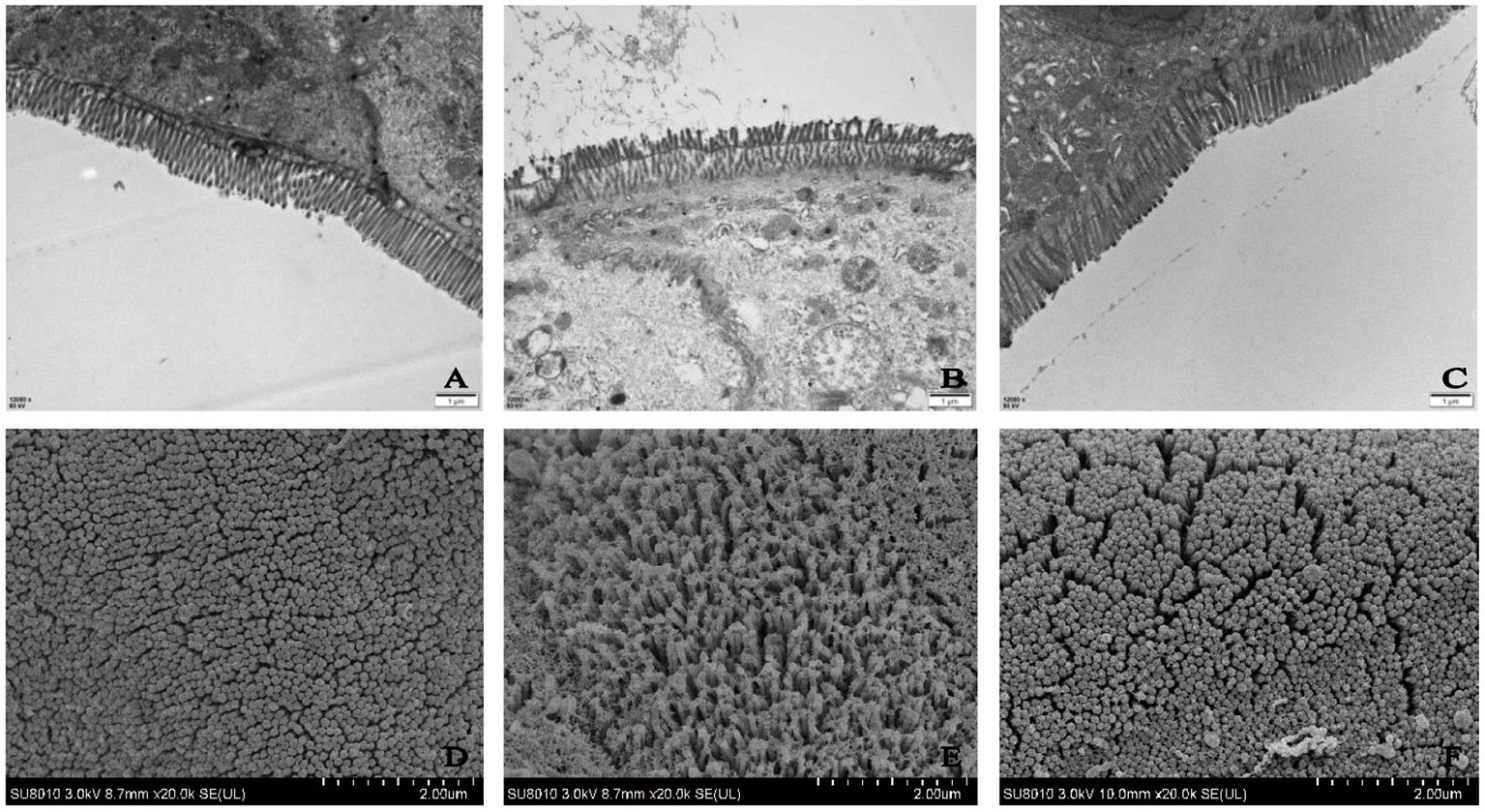
Ultrastructure of mice colon by TEM **(A)** control, **(B)** DSS, **(C)** SP (Magnification times: 12,000x); Ultrastructure of mice colon by SEM **(D)** control, **(E)** DSS, **(F)** SP (Magnification times: 20,000x).

Therefore, the DLP (low-dose SP) group was selected and the ultrastructure of the colonic epithelium was studied to determine the mechanism of SP in alleviating intestinal disorders. TEM and SEM were used to observe the ultrastructural changes in the colonic epithelium at magnifications of 12,000 × and 25,000 × , respectively. TEM revealed that the microvilli in the control (normal) group had the same length and an orderly arrangement (Figures 4A–C). The epithelial cell membrane structure was complete and the tight connection between apical cells was clearly visible. However, the microvilli in the DSS group were obviously damaged, disordered and loose, and were ordered in different directions. Moreover, occasional fractures and indistinct tight connections were observed. After treatment with SP, the microvilli showed an obvious recovery with a more orderly arrangement and slight improvements in the tight connections compared with those in the DSS group. SEM findings further confirmed the repairing effect of SP on the gut (Figures 4D–F). Microvilli in the DSS group were found to be arranged loosely with large gaps, indicating damage to the intestinal mucosal barrier. However, microvilli in the blank and SP groups appeared to have a similar length with a small gap. These results suggested that SP treatment improved the colonic microstructure and morphology in mice with DSS-induced colitis.

### SP Improved Oxidative and Inflammatory Damage in the Serum of Mice With DSS-Induced Colitis

**Figure 5:**
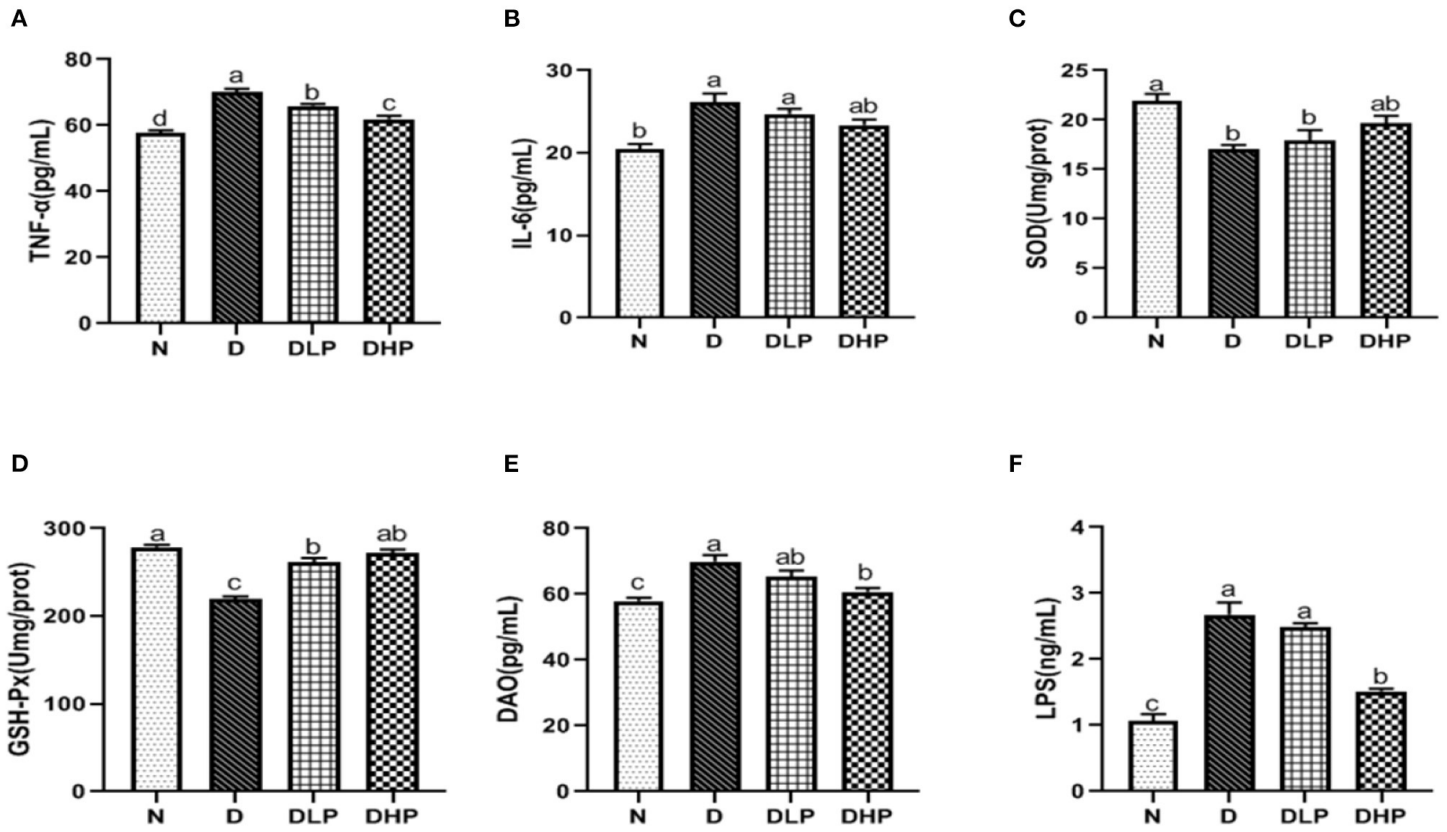
Effect of SP on TNF-α **(A)**, IL-6 **(B)**, SOD **(C)**, GSH-Px **(D)**, DAO **(E)**, and LPS **(F)** in serum of the DSS treated mice. Different letters indicate significant difference between groups (*P* < 0.05).

Inflammatory response, oxidative stress, and damage index of mice with DSS-induced colitis mice were measured using the serum. Serum IL-6 and TNF-α levels were significantly elevated in mice with DSS-induced UC compared with those in the control group (*P* < 0.05), indicating an inflammatory response (Figures 5A,B). On the other hand, serum TNF-α levels were significantly reduced in mice in the low- and high-dose SP groups compared with those in the DSS group (*P* < 0.05). IL-6 levels were less altered after SP intervention, and although a decreasing trend was observed, the changes were not statistically significant when compared with the DSS group. With respect to oxidative stress, SOD and GSH-Px activities were significantly reduced in the serum of mice with DSS-induced UC than that in the normal group (*P* < 0.05). The serum SOD levels of mice in the SP group were not significantly different from those in the DSS group (*P* > 0.05) (Figure 5C). However, serum GSH-Px levels were significantly higher (*P* < 0.05) after treatment with both low-dose and high-dose SP (Figure 5D). In the DSS group (Figures 5E,F), the DAO and LPS levels were significantly higher compared with those in the normal control group. A significant improvement was observed (*P* < 0.05) after SP intervention, especially with the high dose. Collectively, these results suggested that SP had a restorative function in DSS-induced colitis and that a high dose of SP could elicit a better effect.

### SP Suppressed Oxidative and Inflammatory Damage in Colon Tissues of Mice With DSS-Induced Colitis

**Figure 6:**
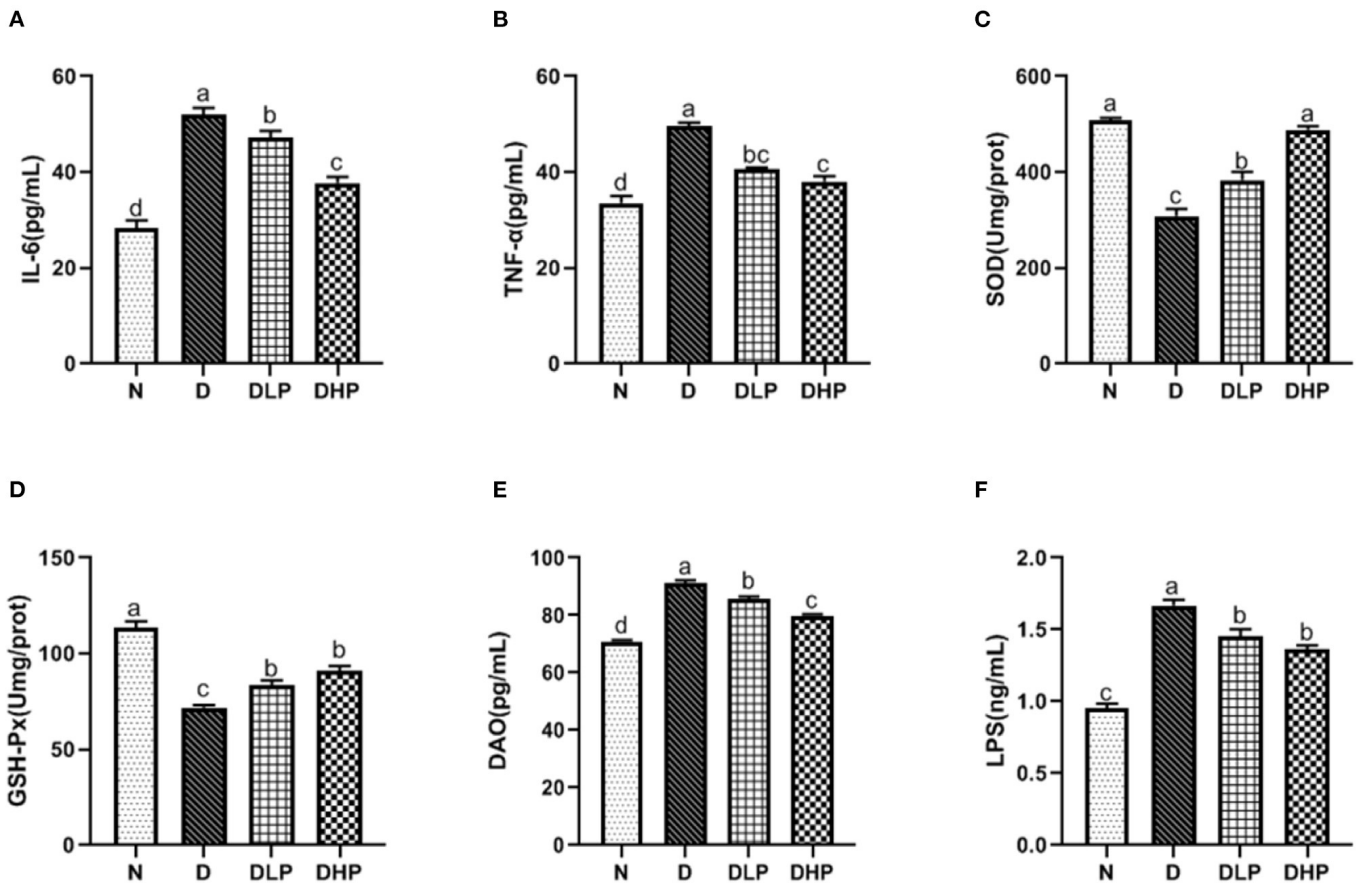
Effect of SP on IL-6 **(A)**, TNF-α **(B)**, SOD **(C)**, GSH-Px **(D)**, DAO **(E)**, and LPS **(F)** in colonic tissue of the DSS treated mice. Different letters indicate significant difference between groups (*P* < 0.05).

We determined the inflammatory response, oxidative stress, and damage index in the colonic tissue of mice with DSS-induced colitis. Levels of the inflammatory factors IL-6 and TNF-α were significantly increased in the colon tissue of mice in the DSS group (*P* < 0.05) compared with those in control mice (Figures 6A,B), which was suggestive of enteritis. Both IL-6 and TNF-α levels in SP-treated mice (*P* < 0.05) were significantly reduced compared with those in mice in the DSS group, indicating the effective regulation of inflammatory factors by SP in the colonic tissue of mice with colitis. Furthermore, SOD and GSH-Px activities were significantly reduced in the colonic tissues of mice in the DSS group compared with those in the normal group (*P* < 0.05) (Figures 6C,D). Treatment with low- and high-dose SP could significantly enhance SOD and GSH-Px activity compared with that in the DSS group (*P* < 0.05), indicating that SP could improve oxidative stress in the mouse model of UC. Findings for the colon damage index (Figures 6E,F) suggested that DAO and LPS levels in the colonic tissues of mice were significantly increased in the DSS group compared with those in the normal group (*P* < 0.05). DAO and LPS levels in the colonic tissues of mice in all SP-treated groups were significantly lower (*P* < 0.05) compared with those of mice in the DSS group, where symptoms of inflammation were observed. This finding suggested that SP had a certain recovery effect in UC mice with intestinal damage.

### SP Regulated Gut Microbiota Composition in Mice With DSS-Induced Colitis

#### α-Diversity Analysis

The detection depth of the intestinal flora is reflected by the coverage. The higher the value, the fewer the undetected sequences in the sample. We found the coverage of the three groups to be >99.9%. α-Diversity analysis was used to evaluate the richness and diversity of the intestinal flora. Species richness of the community within a single sample is reflected by Ace and Chao1, wherein, the larger the number, the higher the richness of the intestinal flora. Shannon and Simpson's indices reflect the diversification of microorganisms, wherein, the larger the value, the richer the diversity. The Shannon index of the DSS group was significantly lower than that of the normal group. After intragastric administration of SP, the Shannon index of the mice increased, but the increase was not significantly different. The results showed no significant differences in the Simpson's, Chao1, and Ace indices among the normal, DSS, and SP-treated groups.

**Figure 7:**
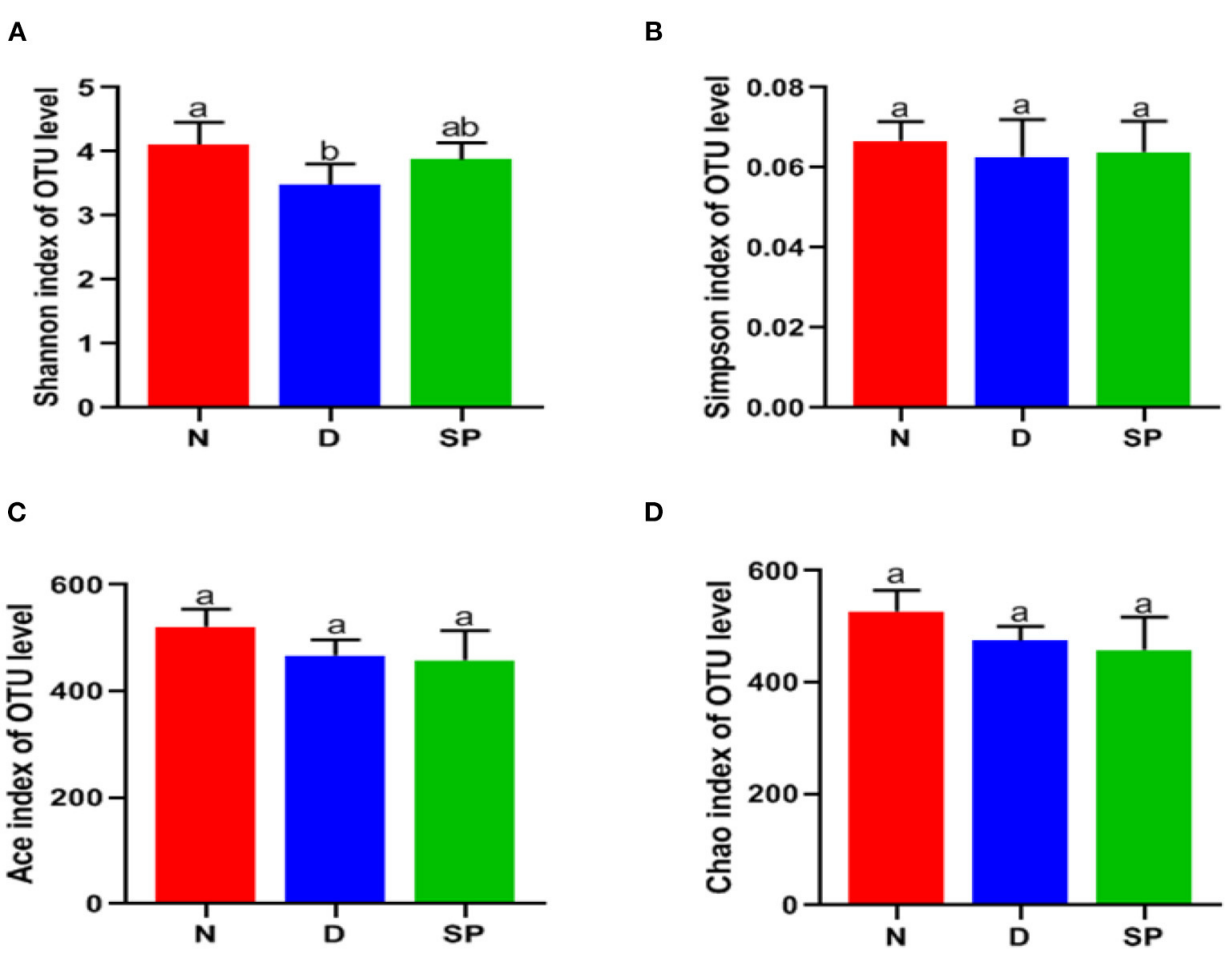
The effects of SP on the alpha diversity of gut microbiota. **(A)** Shannon index, **(B)** Simpson index, **(C)** Ace, **(D)** Chao. N, the control group; D, the DSS-treated group; SP, DSS + 300 mg/kg peptides of *Solenocera crassicornis* by-products.

Experimental results revealed that the species of intestinal flora in the model group was not significantly different compared with that in the control group. However, the species evenness decreased significantly, resulting in a significant reduction in α-diversity in the DSS group. The results are shown in Table 3 and Figure 7.

**Table 3:** Alpha diversity analysis.

**Groups**	**Good's coverage**	**Richness**		**Diversity**	
		**Chao**	**Ace**	**Shannon indices**	**Simpson indices**
N	>99.9%	527.10 ± 46.97^a^	520.16 ± 49.81^a^	4.12 ± 0.34^a^	0.05 ± 0.02^a^
D	>99.9%	485.59 ± 55.42^a^	474.36 ± 55.33^a^	3.49 ± 0.31^b^	0.09 ± 0.04^a^
SP	>99.9%	452.61 ± 69.02^a^	445.14 ± 67.52^a^	3.80 ± 0.30^ab^	0.07 ± 0.03^a^

*Different letters (a,b) suggest p <0.05*.

#### Venn Diagram Analysis

**Figure 8:**
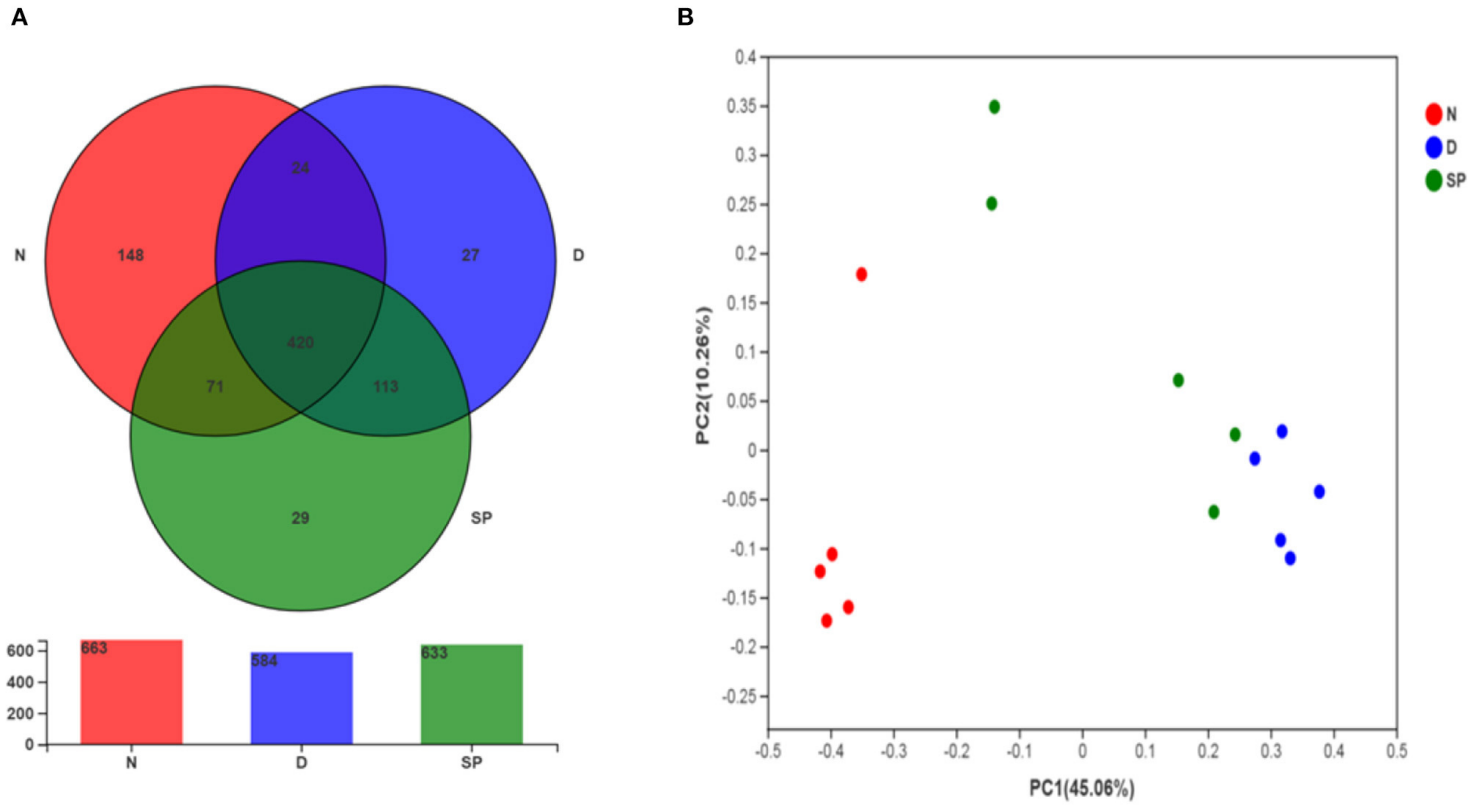
The effect of SP treatment on the diversity and structure of gut microbiota in colitis mice. **(A)** Venn diagram comparison of all the samples. **(B)** Plots of PCoA of gut microbiota in the DSS-induced colitis mice. All results were compared among 3 diet treatment classifications including the control group (N), the DSS-treated group (D), DSS + 300 mg/kg peptides of *Solenocera crassicornis* by-products (SP). Data were presented as mean ± SD (*n* = 5).

Venn diagrams are used to determine the abundances of common and unique species [such as operational taxonomic units (OTUs)] in several species or samples and more clearly indicate the similarities in species (such as OTU) in various environments of a sample. As shown in Figure 8A, the OTUs of the normal, DSS, and SP groups are 663, 584, and 633, respectively, and the number of the same three groups of OTUs is 420. The number of the same OTUs in the normal and SP groups was higher than the same OTUs in the DSS and SP groups, indicating that the intestinal flora of SP-treated mice improved and was more similar to the control group compared with the DSS group.

#### Principal Coordinates Analysis

PCoA is commonly used to determine the differences within and between groups of samples during microbiological evaluation. Figure 8B shows a significant separation of values for model and normal mice, indicating that DSS could considerably change the intestinal flora of mice. Model mice treated with a low dosage of SP (300 mg/kg) had less separation compared with the bacterial composition in normal mice as indicated by the tight clustering on the two-dimensional PCoA plot, which suggested that the differences in intestinal flora between the blank and SP groups were reduced. These results showed the ameliorative and protective effect of SP in DSS-induced colitis.

#### Changes in Intestinal Flora at the Phylum Level

**Figure 9:**
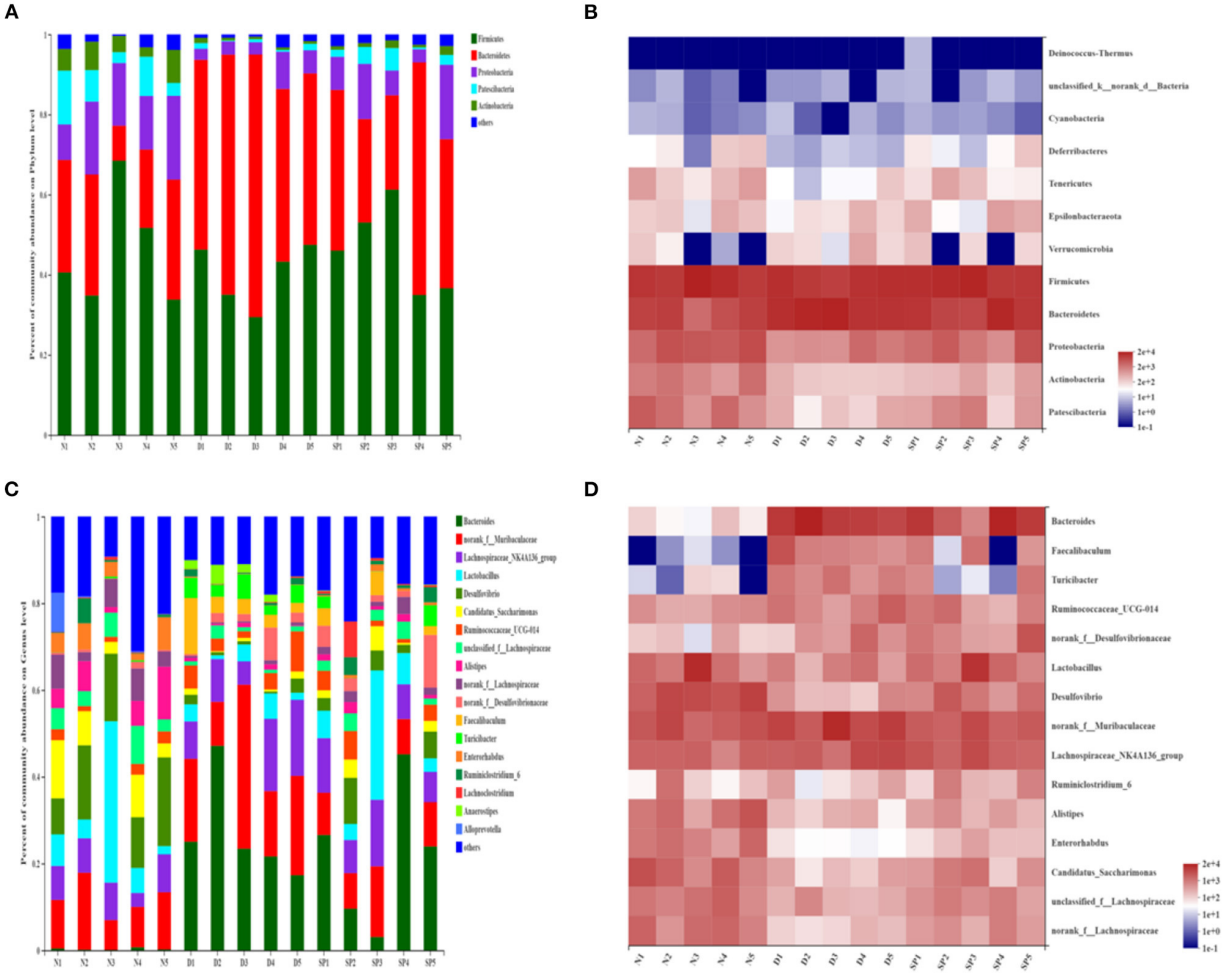
The effect of SP treatment on the changes in gut microbiota in colitis mice. **(A)** The community structures of the observed samples at phylum levels. **(B)** Heat map of all the samples on phylum levels. **(C)** The community structures of the observed samples at genus levels. **(D)** Heat map of all the samples on genus levels. Data were presented as mean ± SD (*n* = 5).

After analysis at the phylum level based on the histogram, the composition of the intestinal bacteria phyla was determined to mainly comprise *Bacteroidetes, Firmicutes, Proteobacteria, Patescibacteria*, and *Actinobacteria*, of which *Firmicutes* and *Bacteroidetes* accounted for the largest proportion. The average proportions of *Bacteroidetes* in the normal, DSS, and SP-treated groups were 23.31, 51.76, and 36.95%, respectively, and the average proportion of *Firmicutes* was 45.88, 40.29, and 46.38%, respectively. It can be seen from the heat map that the abundance of *Firmicutes* was decreased in the DSS group compared with that in the normal group, whereas the abundance of *Bacteroidetes* had increased significantly (*P* < 0.05). Furthermore, the abundances of *Proteobacteria, Patescibacteria, Tenericutes*, and *Actinobacteria* had also decreased significantly (*P* < 0.05). However, compared with that in the DSS group, the expansion of *Bacteroidetes* induced by DSS treatment was suppressed in the SP group, and the abundance of *Firmicutes* in mice treated with SP had increased. In particular, the abundances of *Proteobacteria, Patescibacteria, Tenericutes*, and *Actinobacteria* in the SP-treated groups were reversed compared with the DSS group and were close to that of the normal group (Figures 9A,B).

The results suggested that the composition of the intestinal flora was significantly damaged and that the balance of the original flora was disrupted in modeled mice. SP could inhibit the damage caused by pathogenic bacteria and maintain the intestinal flora in a healthy state. Moreover, it created a beneficial biological environment for restoring the balance of the structural composition of intestinal flora.

#### Changes in Intestinal Flora at the Genus Level

At the genus level (Figures 9C,D), the main bacterial genera were determined to be *Bacteroides, Muribaculaceae, Lachnospiraceae, Lactobacillus, Desulfovibrio, Candidatus_Saccharimonas, Alistipes*, and *Ruminococcaceae*. Among them, the average proportion of *Lactobacillus* in the normal, DSS, and SP groups was 11.29, 3.25, and 10.03%, respectively, and the average proportion of *Bacteroides* was 0.3, 26.90, and 21.67%, respectively. Heat map analysis of the genus revealed that the abundances of *Bacteroides, Faecalibaculum*, and *Turicibacter* in the intestines of DSS-modeled mice were significantly increased (*P* < 0.05), whereas the abundances of *Desulfovibrio, Lactobacillus, Lachnospiraceae, Alistipes*, and *Enterorhabdus* were lower than those in the control group (*P* < 0.05). SP intervention could reverse this abundance.

## Discussion

UC is an inflammatory bowel disease that usually affects certain regions of the colon uninterruptedly or damages the entire colon in severe cases. The inflammation is generally confined to the mucosa (23) and this condition is likely linked to immune dysregulation of the intestine. Amongst the immunoregulatory factors, oxidative stress is one of the main triggers responsible for the continuous development of the disease (24). Moreover, diet is closely linked to physical health and has shown to have great potential in the prevention of UC. Studies have shown that SP has many biological activities such as antioxidant, anti-inflammatory, and antibacterial effects. Thus, in this study, we isolated a new type of SP (MW distribution was mainly below 1,000 Da and the essential amino acid content accounted for 54.50%) from Chinese pipe whip shrimp (*S. crassicornis*) and determined its effects on oxidative stress, intestinal microbiota, intestinal barrier, and inflammation response *in vivo* in mice with DSS-induced colitis.

The antioxidant system in organisms is mainly composed of two components, namely, the oxidant and antioxidant enzymes. Collectively, this system can effectively scavenge oxygen free radicals and stabilize the cell membrane. SOD and GSH-Px together form a defense system to scavenge free radicals. In UC, the antioxidant system is damaged due to ROS release, and serum SOD and GSH-Px levels are reduced, which damages the intestinal mucosa and ultimately leads to intestinal inflammation (25). Moreover, among the inflammatory factors, oxidative stress plays an important role in the pathogenesis of IBD (26). Colitis can be prevented by inhibiting the biological processes of oxidative stress and the related damage to DNA, lipids, and proteins (27). In this study (Figures 5, 6), SOD and GSH-Px levels in the colonic tissues and serum of mice in the DSS group were significantly lower than those in normal mice (*P* < 0.05), indicating that the antioxidant system was compromised in mice with UC. SOD and GSH-Px levels in the colon tissue and serum of mice are increased after treatment with different doses of SP compared with that in the DSS group of mice, suggesting that SP could improve the antioxidant capacity of cells and prevent damage by UC and intestinal oxidative stress by upregulation of the antioxidant enzymes. Although the pathogenesis of UC has not been elucidated, an imbalance in the anti-inflammatory (IL-10) and pro-inflammatory cytokines (IL-6 and TNF-α) is attributed to regulating the inflammatory response (28). Studies have reported that IL-6 and TNF-α levels are significantly increased in mice with DSS-induced colitis and that these components are involved in the regulation of signaling pathways such as apoptosis, migration, and protein synthesis (29, 30). TNF-α can activate neutrophils and cause microcirculation disorders in the intestinal mucosa. The STAT3 signaling pathway can be activated by IL-6 and plays a significant regulatory role in the pathogenesis of UC (31). Our results showed the SP treatment could reduce IL-6 and TNF-α levels in the serum and colon of mice with UC. IL-6 and TNF-α levels in the serum and colonic tissues were higher in mice in the model group than those in the blank group. SP treatment significantly reduced IL-6 levels (*P* < 0.05) in the serum and colon as well as TNF-α levels in the colon significantly (*P* < 0.05) compared with those in the model group, indicating that SP treatment could reduce the release of inflammatory factors and protect from damage caused by UC. Additionally, DAO and LPS levels in the intestine and serum reflect disruption of the intestinal mechanical barrier (32). Immunogenic endotoxins such as LPS are produced by the intestinal microbiota and released from the gut into the blood, exacerbating the inflammatory response during circulation (20). DAO is a strongly reactive intracellular enzyme present in mammalian intestinal epithelial cells and is released into the intestinal tract and bloodstream as the intestinal epithelial cells are stripped and begin to disintegrate (20). In this study, DAO and LPS levels in the colon and serum of mice in all SP-treated groups decreased significantly compared with those in the model group (*P* < 0.05), indicating that SP could reduce intestinal mucosal damage and help restore the intestinal mucosal barrier. Furthermore, we determined the ultrastructure of the colon of mice with UC using HE staining, TEM, and SEM. The microvilli of colon tissues from the SP group showed obvious recovery with a more orderly arrangement and faint visibility of the tight connections (Figures 3, 4). Besides, the length of the microvilli and the small gap in tissues from SP-treated mice were similar to those observed in the tissues of mice from the normal group (Figure 4). These changes resulted from the combined effect of changes in oxidative stress and levels of intracellular enzymes, endotoxin, and inflammatory factors. Interestingly, the results of the experiment showed that the low-dose SP group had more significant effects than the high-dose SP group in terms of colonic morphology and body weight, while the high-dose SP group was better in terms of inflammatory and antioxidant indices. This may be related to the dose and time of administration and intestinal absorption capacity. The short administration time and slow intestinal absorption resulted in worse inflammatory and antioxidant effects in the low-dose SP than in the high-dose SP, but better than in the model group. In contrast, low-dose SP may be less irritating to the intestine than high-dose SP, promoting intestinal absorption in a gentler manner, achieving therapeutic effects while better maintaining colon morphology and allowing increased body weight.

Increasing evidence shows the role of intestinal flora in the prevention of UC during drug therapy (33). UC is associated with gut microbiota dysbiosis (the inflammatory state leads to an imbalance in the gut microbiota mainly through intestinal oxidation and alteration of the metabolic environment), which leads to an increase in blood LPS levels and intestinal mucosa permeability. Thus, we determined the effects of SP on the diversity and composition of gut microbiota in mice with UC. In this study, we analyzed the effect of SP on the intestinal flora of mice with UC using 16S rRNA gene sequencing. These results revealed a significant disruption in the balance of intestinal flora in mice with UC, which was primarily manifested by a reduction in α-diversity. We found that the Chao 1, Ace, and Shannon indices were lower in the SP-treated groups than in the model group (Figure 7), which suggested that the α-diversity of gut flora was significantly reduced after SP treatment. This change might be associated with the antibacterial activity of SP, as reported by Chen et al. (34). To further determine the regulatory impact of SP on gut flora, we studied the microbial composition using a Venn diagram and PCoA. Our results indicated that the total number of OTUs was significantly lower in the DSS group, which was less than the normal group in total OTUs. Moreover, the number of OTUs in the SP group was closer to that in the normal group, which might be explained on the basis that DSS treatment decreased the related beneficial bacteria and SP treatment attenuated the above bacterial species until they returned to normal conditions (based on the Venn diagram, the number of the same OTUs in the normal and SP groups was higher than the same OTUs in the DSS and SP groups) (Figure 8A). Besides, based on PCoA (Figure 8B), we arrived at a similar conclusion that there was an obvious separation between the control and DSS groups, whereas the SP group showed less separation between the bacterial members compared with that in the normal group.

*Bacteroidetes* and *Firmicutes* are the primary phyla that species that make up the intestinal microbiota in several organisms in addition to other important phyla in the gut, such as *Proteobacteria, Patescibacteria, Tenericutes*, and *Actinobacteria* (35, 36). This abundant microbiota provides ample genetic resources to the host, including essential vitamins, necessary energy, and an environment for intestinal development (37). The relative abundance of *Bacteroidetes* in the gut of mice with DSS-induced colitis was higher than that in the normal group, whereas the relative abundance of *Firmicutes* was comparatively lower. Our findings were consistent with the results of the previous studies that report the imbalance in the gut microbiota in colitis (38). SP intervention restored the *Firmicutes*/*Bacteroidetes* ratio to almost normal. A previous study suggests that the *Firmicutes*/*Bacteroidetes* ratio in the gut may affect the production of short-chained fatty acids (SCFAs), thereby regulating the weight and inhibiting an inflammatory response (39). Correlation with the gut microbiota was analyzed in-depth at the genus level in *Bacteroides*. It has been reported that the relative abundance of *Bacteroides* is positively correlated with pro-inflammatory cytokines; furthermore, a reduction in the abundance of *Bacteroides* has a protective effect in IBD (40, 41). We found that the abundance of the pathogenic *Bacteroides* increased significantly in mice with DSS-induced colitis, which was in accordance with previous investigations (42, 43). Moreover, our study showed that SP could significantly inhibit the proliferation of such harmful bacteria. The probiotic *Lactobacillus* is found in daily foods, mostly in yogurt, and has a regulatory effect on the human gut. *Lactobacillus* can alleviate the clinical symptoms of IBD and maintain intestinal balance. SCFA-producing bacteria (such as *Alistipes*) are associated with the alleviation of intestinal inflammation, which can promote intestinal maturation (44). Our findings indicated that the relative abundance of *Lactobacillus* and *Alistipes* in mice with UC was lower than that in normal mice. However, SP intervention inhibited the reduction of *Lactobacillus* and *Alistipes* in the gut of mice with UC. These findings were consistent with a study that reported the efficacy of certain peptides from egg white (45) and microcin (46) in alleviating DSS-induced UC. A previous study has shown that DSS treatment not only significantly decreases the abundance of *Desulfovibrio, Lachnospiraceae*, and *Enterorhabdus*, but also increases the abundance of *Faecalibaculum* and *Tericibacter* (47). In the current study, SP treatment was found to reverse the above phenomenon.

Overall, our findings indicated the beneficial and regulatory role of SP on gut flora. Thus, SP could maintain the structural stability of gut flora in mice with UC by regulating the increase in beneficial intestinal bacteria and the decrease in pathogenic bacteria, thereby creating a beneficial biological environment to restore the abundance of intestinal flora to a normal, balanced state.

## Conclusion

In summary, the findings from our study suggest that SP could effectively alleviate the pathological damage and clinical symptoms, significantly improve oxidative stress and the inflammatory response, and protect the intestinal barrier of mice with UC. Moreover, SP intervention could inhibit functional disorders and improve the structural components of intestinal flora in mice with UC. However, the causal relationship between SCFA production and intestinal inflammation is unclear and needs further investigation. Therefore, our findings suggest the potential of SP in the management and treatment of UC.

## Data Availability Statement

The datasets presented in this study can be found in online repositories. The names of the repository/repositories: Genbank (NCBI) BioProject, and PRJNA774469.

## Ethics Statement

All experiments involving animal use were performed with the approval of the Institutional Animal Care and Use Committee at Zhejiang Ocean University (Approval No. 2019003).

## Author Contributions

XX, LC, YC, and BZ conceived and designed the experiments. QJ, WS, and JL performed the main experiments and analyzed the data. YZ and SD helped the project administration. XX, QJ, and YC prepared the figures and wrote the paper. All authors contributed to the article and approved the submitted version.

## Funding

This work was supported by grants from Zhejiang Province Public Welfare Technology Application Research Project (LGJ21C20001) and Zhejiang Provincial Key Research and Development Project of China (2019C02076 and 2019C02075).

## Conflict of Interest

The authors declare that the research was conducted in the absence of any commercial or financial relationships that could be construed as a potential conflict of interest.

## Publisher's Note

All claims expressed in this article are solely those of the authors and do not necessarily represent those of their affiliated organizations, or those of the publisher, the editors and the reviewers. Any product that may be evaluated in this article, or claim that may be made by its manufacturer, is not guaranteed or endorsed by the publisher.

## References

[1] Deng Z Cui C Wang Y Ni J Zheng L Wei HK. FSGHF3 and peptides, prepared from fish skin gelatin, exert a protective effect on DSS-induced colitis via the Nrf2 pathway. Food Funct. (2020) 11:414–23. 10.1039/C9FO02165E 31825438

[2] Schoultz I Keita AV. Cellular and molecular therapeutic targets in inflammatory bowel disease-focusing on intestinal barrier function. Cells. (2019) 8:193. 10.3390/cells8020193 30813280 PMC6407030

[3] Tian T Wang Z Zhang J. Pathomechanisms of oxidative stress in inflammatory bowel disease and potential antioxidant therapies. Oxidat Med Cell Longevity. (2017) 2017:1–18. 10.1155/2017/4535194 28744337 PMC5506473

[4] Shi C Yue F Shi F Qin Q Wang L Wang G. Selenium-containing amino acids protect dextran sulfate sodium-induced colitis via ameliorating oxidative stress and intestinal inflammation. J Inflamm Res. (2021) 14:85–95. 10.2147/JIR.S288412 33488110 PMC7814278

[5] Qin M Qiu Z. Changes in TNF-α, IL-6, IL-10 and VEGF in rats with ARDS and the effects of dexamethasone. Exp Therap Med. (2018) 17:383–7. 10.3892/etm.2018.6926 30651808 PMC6307422

[6] Guo C Wang Y Zhang S Zhang X Ding K. Crataegus pinnatifida polysaccharide alleviates colitis via modulation of gut microbiota and SCFAs metabolism. Int J Biol Macromol. (2021) 181:357–68. 10.1016/j.ijbiomac.2021.03.137 33774071

[7] Cui L Guan X Ding W Luo Y Feng L. Scutellaria baicalensis Georgi polysaccharide ameliorates DSS-induced ulcerative colitis by improving intestinal barrier function and modulating gut microbiota. Int J Biol Macromol. (2020) 166:1035–45. 10.1016/j.ijbiomac.2020.10.259 33157130

[8] Sun J Chen H Kan J Gou Y Liu J Zhang X. Anti-inflammatory properties and gut microbiota modulation of an alkali-soluble polysaccharide from purple sweet potato in DSS-induced colitis mice. Int J Biol Macromol. (2020) 153:708–22. 10.1016/j.ijbiomac.2020.03.053 32169445

[9] Zong X Hu W Song D Li Z Du H Lu Z. Porcine lactoferrin-derived peptide LFP-20 protects intestinal barrier by maintaining tight junction complex and modulating inflammatory response. Biochem Pharmacol. (2016) 104:74–82. 10.1016/j.bcp.2016.01.009 26776304

[10] Zhang L Wei X Zhang R Petitte JN Si D Li Z. Design and development of a novel peptide for treating intestinal inflammation. Front Immunol. (2019) 10:1841. 10.3389/fimmu.2019.01841 31447849 PMC6691347

[11] Zhang Y Chen S Zong X Wang C Shi C Wang F. Peptides derived from fermented soybean meal suppresses intestinal inflammation and enhances epithelial barrier function in piglets. Food Agric Immunol. (2020) 31:120–35. 10.1080/09540105.2019.1705766

[12] Song R Jia Z Xu Y Zhang X Wei R Sun J. Saponification to improve the antioxidant activity of astaxanthin extracts from *Penaeus sinensis* (*Solenocera crassicornis*) by-products and intervention effect on Paracetamol-induced acute hepatic injury in rat. J Funct Foods. (2020) 73:104150. 10.1016/j.jff.2020.104150

[13] An XH Lv Y Li WG Liang JG Xu CH Zhang C-x. Antimicrobial peptides purified from *Penus chinensis*. Zool Res. (2005) 26:410–15.

[14] Jiang S Zhang Z Yu F Zhang Z Yang Z Tang Y. Ameliorative effect of low molecular weight peptides from the head of red shrimp (Solenocera crassicornis) against cyclophosphamide-induced hepatotoxicity in mice. J Funct Foods. (2020) 72:104085. 10.1016/j.jff.2020.104085

[15] Jiang S Zhang Z Huang F Yang Z Yu F Tang Y. Protective Effect of Low Molecular Weight Peptides fromSolenocera crassicornisHead against Cyclophosphamide-Induced Nephrotoxicity in Miceviathe Keap1/Nrf2 Pathway. Antioxidants. (2020) 9:745. 10.3390/antiox9080745 32823691 PMC7465301

[16] Binsan W Benjakul S Visessanguan W Roytrakul S Tanaka M Kishimura H. Antioxidative activity of Mungoong, an extract paste, from the cephalothorax of white shrimp (Litopenaeus vannamei). Food Chem. (2008) 106:185–93. 10.1016/j.foodchem.2007.05.065

[17] Lin MC Pan CY Hui CF Chen JY Wu JL. Shrimp anti-lipopolysaccharide factor (SALF), an antimicrobial peptide, inhibits proinflammatory cytokine expressions through the MAPK and NF-κB pathways in LPS-induced HeLa cells. Peptides. (2013) 40:42–8. 10.1016/j.peptides.2012.11.010 23247147

[18] Liu H Wang J Mao Y Liu M Niu S-f Qiao Y. Identification and expression analysis of a novel stylicin antimicrobial peptide from Kuruma shrimp (*Marsupenaeus japonicus*). Fish Shellfish Immunol. (2015) 47:817–23. 10.1016/j.fsi.2015.09.044 26439413

[19] Arockiaraj J Kumaresan V Bhatt P Palanisamy R Gnanam AJ Pasupuleti M. A novel single-domain peptide, anti-LPS factor from prawn: synthesis of peptide, antimicrobial properties and complete molecular characterization. Peptides. (2014) 53:79–88. 10.1016/j.peptides.2013.11.008 24269604

[20] Zhou X Xiang X Zhou Y Zhou T Deng S Zheng B. Protective effects of Antarctic krill oil in dextran sulfate sodium-induced ulcerative colitis mice. J Funct Foods. (2021) 79:104394. 10.1016/j.jff.2021.104394

[21] Danielsen EM De Haro Hernando A Yassin M Rasmussen K Olsen J Hansen GH. Short-term tissue permeability actions of dextran sulfate sodium studied in a colon organ culture system. Tissue Barriers. (2020) 8:1728165. 10.1080/21688370.2020.1728165 32079482 PMC7549740

[22] Chen Y Jin L Li Y Xia G Chen C Zhang Y. Bamboo-shaving polysaccharide protects against high-diet induced obesity and modulates the gut microbiota of mice. J Funct Foods. (2018) 49:20–31. 10.1016/j.jff.2018.08.015

[23] Nielsen TS Fredborg M Theil PK Yue Y Bruhn LV Andersen V. Dietary red meat adversely affects disease severity in a pig model of DSS-induced colitis despite reduction in colonic pro-inflammatory gene expression. Nutrients. (2020) 12:1728. 10.3390/nu12061728 32526985 PMC7353045

[24] Ingawale DK Mandlik SK Patel SS. Hecogenin and fluticasone combination attenuates TNBS-induced ulcerative colitis in rats via downregulation of pro-inflammatory mediators and oxidative stress. Immunopharmacol Immunotoxicol. (2021) 43:160–70. 10.1080/08923973.2021.1872617 33435764

[25] Canbakan B Koroglu E Atay K Tuncer M Senturk H. Assessment of oxidative stress parameters for the disease activity evaluation in patients with ulcerative colitis. J Crohns Colitis. (2016) 10:S173. 10.1093/ecco-jcc/jjw019.284

[26] Goyal N Rana A Ahlawat A Bijjem K Kumar P. Animal models of inflammatory bowel disease: a review. Inflammopharmacology. (2014) 22:219–33. 10.1007/s10787-014-0207-y 24906689

[27] Zhao H Cheng N Zhou W Chen S Cao W. Honey polyphenols ameliorate DSS-induced ulcerative colitis via modulating gut microbiota in rats. Mol Nutr Food Res. (2019) 63:1900638. 10.1002/mnfr.201900638 31533201

[28] Wang Z Xue W Wang CL Wang L Chen S Zhang DB. Tryptanthrin protects mice against dextran sulfate sodium-induced colitis through inhibition of TNF-α/NF-κB and IL-6/STAT3 pathways. Molecules. (2018) 23:1062. 10.3390/molecules23051062 29724065 PMC6099556

[29] Do EJ Hwang SW Kim SY Ryu YM Cho EA Chung EJ. Suppression of colitis-associated carcinogenesis through modulation of IL-6/STAT3 pathway by balsalazide and VSL#3. J Gastroenterol Hepatol. (2016) 31:1453–61. 10.1111/jgh.13280 26711554

[30] Choi JH Chung KS Jin BR Cheon SY Nugroho A Roh SS. Anti-inflammatory effects of an ethanol extract of Aster glehni via inhibition of NF-κB activation in mice with DSS-induced colitis. Food Funct. (2017) 8:2611–20. 10.1039/C7FO00369B 28695925

[31] Pandurangan AK Mohebali N Hasanpourghadi M Chung YL Mustafa MR Esa NM. Boldine suppresses dextran sulfate sodium-induced mouse experimental colitis: NF-κB and IL-6/STAT3 as potential targets. Biofactors. (2016) 42:247–58. 10.1002/biof.1267 26891685

[32] Wen ZS u MD Tang Z Zhou TY Zhang ZS Song HH. Low molecular seleno-aminopolysaccharides protect the intestinal mucosal barrier of rats under weaning stress. Int J Mol Sci. (2019) 20:5727. 10.3390/ijms20225727 31731602 PMC6888692

[33] Eom T Kim YS Choi CH Sadowsky MJ Unno T. Current understanding of microbiota- and dietary-therapies for treating inflammatory bowel disease. J Microbiol. (2018) 56:189–98. 10.1007/s12275-018-8049-8 29492876

[34] Chen M Liao Z Lu B Wang M Lin L Zhang S. Huang-Lian-Jie-Du-Decoction ameliorates hyperglycemia and insulin resistant in association with gut microbiota modulation. Front Microbiol. (2018) 9:2380. 10.3389/fmicb.2018.02380 30349514 PMC6186778

[35] Fabrega M-J Rodriguez-Nogales A Garrido-Mesa J Algieri F Badia J Gimenez R. Intestinal anti-inflammatory effects of outer membrane vesicles from escherichia coli nissle 1917 in DSS-experimental colitis in mice. Front Microbiol. (2017) 8:1274. 10.3389/fmicb.2017.01274 28744268 PMC5504144

[36] Makki K Deehan EC Walter J Bäckhed F. The impact of dietary fiber on gut microbiota in host health and disease. Cell Host Microbe. (2018) 23:705–15. 10.1016/j.chom.2018.05.012 29902436

[37] Shin N-R Whon TW Bae J-W. Proteobacteria: microbial signature of dysbiosis in gut microbiota. Trends Biotechnol. (2015) 33:496–503. 10.1016/j.tibtech.2015.06.011 26210164

[38] Franzosa EA Sirota-Madi A Avila-Pacheco J Fornelos N Haiser HJ Reinker S. Gut microbiome structure and metabolic activity in inflammatory bowel disease. Nat Microbiol. (2019) 4:293. 10.1038/s41564-018-0306-4 30531976 PMC6342642

[39] Mcilroy J Ianiro G Mukhopadhya I Hansen R Hold GL. Review article: the gut microbiome in inflammatory bowel disease—avenues for microbial management. Aliment Pharmacol Ther. (2018) 47:26–42. 10.1111/apt.14384 29034981

[40] Su L Mao C Wang X Li L Tong H Mao J. The anti-colitis effect of schisandra chinensis polysaccharide is associated with the regulation of the composition and metabolism of gut microbiota. Front Cell Infect Microbiol. (2020) 10:519479. 10.3389/fcimb.2020.519479 33194780 PMC7609416

[41] Zhang Y Tan L Li C Wu H Ran D Zhang Z. Sulforaphane alter the microbiota and mitigate colitis severity on mice ulcerative colitis induced by DSS. AMB Express. (2020) 10:119. 10.1186/s13568-020-01053-z 32621086 PMC7334330

[42] Wu X Wang L Tang L Wang L Cao S Wu Q. Salvianolic acid B alters the gut microbiota and mitigates colitis severity and associated inflammation. J Funct Foods. (2018) 46:312–19. 10.1016/j.jff.2018.04.068

[43] Lin Y Zheng X Chen J Luo D Xie J Su Z. Protective effect of Bruguiera gymnorrhiza (L.) Lam. Fruit on dextran sulfate sodium-induced ulcerative colitis in mice: role of Keap1/Nrf2 pathway and gut microbiota. Front Pharmacol. (2020) 10:1602. 10.3389/fphar.2019.01602 32116661 PMC7008401

[44] Walker A Pfitzner B Harir M Schaubeck M Calasan J Heinzmann SS. Sulfonolipids as novel metabolite markers of Alistipes and Odoribacter affected by high-fat diets. Sci Rep. (2017) 7:11047. 10.1038/s41598-017-10369-z 28887494 PMC5591296

[45] Ge H Cai Z Chai J Liu J Liu B Yu Y. Egg white peptides ameliorate dextran sulfate sodium-induced acute colitis symptoms by inhibiting the production of pro-inflammatory cytokines and modulation of gut microbiota composition. Food Chem. (2021) 360:129981. 10.1016/j.foodchem.2021.129981 34020366

[46] Shang L Yu H Liu H Chen M Zeng X Qiao S. Recombinant antimicrobial peptide microcin J25 alleviates DSS-induced colitis via regulating intestinal barrier function and modifying gut microbiota. Biomed Pharmacother. (2021) 139:111127. 10.1016/j.biopha.2020.111127 33819810

[47] Yang B Chen H Gao H Wang J Stanton C Ross RP. Bifidobacterium breve CCFM683 could ameliorate DSS-induced colitis in mice primarily via conjugated linoleic acid production and gut microbiota modulation. J Funct Foods. (2018) 49:61–72. 10.1016/j.jff.2018.08.014

